# Dark eyes in female sand gobies indicate readiness to spawn

**DOI:** 10.1371/journal.pone.0177714

**Published:** 2017-06-07

**Authors:** Karin H. Olsson, Sandra Johansson, Eva-Lotta Blom, Kai Lindström, Ola Svensson, Helen Nilsson Sköld, Charlotta Kvarnemo

**Affiliations:** 1 Department of Biological and Environmental Sciences, University of Gothenburg, Gothenburg, Sweden; 2 Environmental and Marine Biology, Åbo Akademi University, Turku, Finland; 3 Centre for Marine Evolutionary Biology, University of Gothenburg, Gothenburg, Sweden; 4 Sven Lovén Center for Marine Infrastructure Kristineberg, University of Gothenburg, Gothenburg, Sweden; Glasgow Caledonian University, UNITED KINGDOM

## Abstract

In animals, colorful and conspicuous ornaments enhance individual attractiveness to potential mates, but are typically tempered by natural selection for crypsis and predator protection. In species where males compete for females, this can lead to highly ornamented males competing for mating opportunities with choosy females, and vice versa. However, even where males compete for mating opportunities, females may exhibit conspicuous displays. These female displays are often poorly understood and it may be unclear whether they declare mating intent, signal intrasexual aggression or form a target for male mate preference. We examined the function of the conspicuous dark eyes that female sand gobies temporarily display during courtship by experimentally testing if males preferred to associate with females with artificially darkened eyes and if dark eyes are displayed during female aggression. By observing interactions between a male and two females freely associating in an aquarium we also investigated in which context females naturally displayed dark eyes. We found that dark eyes were more likely to be displayed by more gravid females than less gravid females and possibly ahead of spawning, but that males did not respond behaviorally to dark eyes or prefer dark-eyed females. Females behaving aggressively did not display dark eyes. We suggest that dark eyes are not a signal per se but may be an aspect of female mate choice, possibly related to vision.

## Introduction

Ornaments, whether in the form of colors, long feathers, fins, or combs, are often shown predominantly by one of the sexes, and often only during the reproductive season. When this is the case, sexual selection is a likely explanation for the evolution of the ornament [1,2]. Yet, evolutionary explanations for female ornamentation have long been debated. Explanations often include correlated gene expression, that is, when an ornament expressed by males is also shown by females, but to a lesser degree, simply because they carry the same genes [3–5]. However, in cases when only females show an ornament, this explanation becomes irrelevant.

Another explanation is that an ornament may function as a declaration of intent by females. Prefacing a communication or a behavior by some form of attention-seeking display to command the attention of the desired receivers has been shown both empirically [6] and theoretically [7] to enhance the reliability of the communication. In fish, female lagoon gobies *Knipowitschia panizzai* display a prominent yellow spot on the belly just before spawning that correlates to current fecundity [8]. For an ornament to serve as a declaration of intent, it needs to be plastic and most pronounced when females are ready to mate.

A third explanation is that female ornamentation results from female-female competition [9,10]. Such intra-sexual competition can be for mating opportunities. For example, in the sex-role reversed pipefish *Syngnathus typhle*, ornamented females compete for matings [11]. Larger females are more fecund and can dominate smaller females, even to the extent that small females postpone reproduction and invest in growth instead [12]. Intra-sexual competition may also concern resources that directly or indirectly affect the competitors’ ability to reproduce. In *Eclectes roratus* parrots, for example, females have bright blue and red plumage while males are green. In this species, females do not compete for males but for nesting holes, which is a limited but crucial resource. Males, in turn, compete for females with nesting holes [13]. LeBas [9] argues that female ornamentation is more likely to evolve in such competitive contexts, than from male mate choice, but the ornaments may still be used by males in mate choice, if female competitive ability correlates with direct or indirect benefits (e.g. fecundity or viability genes).

A fourth explanation for female ornamentation is that it has evolved as a result of male preferences for such a trait [5,14,15]. This phenomenon has been primarily studied in species where the operational sex ratio is female-biased [11,16]. In the dance fly *Rhamphomyia tarsata*, females have structures on their legs known as combs, which males lack. Males, who provide females with a nuptial gift when mating, prefer to mate with females that have long combs and females compete for matings with these choosy males [17]. Similarly, in the pipefish *Nerophis ophidion*, females develop skinfolds and iridescent blue stripes along their bodies during the breeding season and males prefer to mate with more ornamented females. In this species females compete for matings with males that provide care by carrying the developing embryos attached to their skin [11,18,19]. More recently, however, there is increased awareness that male mate choice is not limited to situations with female-biased operational sex ratios, but can be pronounced also in the face of male-biased operational sex ratios [20].

Finally, it is possible that bright coloration in females is not an aspect of sexual communication *per se*, and is therefore not an ornament, but has a different function. Although conspicuous coloration may increase the risk of predation [21,22], certain color patterns such as spots, stripes and so-called eye stripes (a pigmented band through the eye that disrupts the eye contour) may, conversely, improve camouflage [23,24]. However, if a specific coloration efficiently increases camouflage, it would be expected to be displayed by males and females alike. As regards coloration around the eyes in particular, dark markings have also been linked to species inhabiting habitats characterized by shifting light conditions and may serve an anti-glare function [25,26]. Thus, darker eye color could improve the visual accuracy of e.g. female mate choice, while carrying no information to the male.

The sand goby *Pomatoschistus minutus* offers an interesting opportunity to study the phenomenon of limited female coloration. In this species, males adopt a nuptial coloration during the breeding season. Males also construct nests and try to attract females to spawn in their nests by courting. During such male courtship, females often court the male back, and may display conspicuous dark coloration around the eyes [27–29], which males do not. This is noteworthy because the sand goby is a species with conventional sex roles, that is, males compete for mating opportunities, females are the more choosy sex and the operational sex ratio varies over the breeding season from parity to male-biased [30–32]. Still, female-female interactions are common, especially under experimentally manipulated female-biased sex ratio [27], and the presence of other females contributes to speedier female mate choice [33]. Moreover, under experimental conditions males can be seen to deny females access to their nests by blocking the entrance (e.g. this study, and personal observation, KHO, OS). Thus, some degree of male mate choice seems plausible. Fish typically change color by translocating pigmented organelles inside chromatophore cells in the skin and the eyes [34,35], which can make coloration highly plastic. Consistent with this, female sand gobies are able to flexibly adopt and again lose their dark eye coloration, sometimes gradually, other times quickly, even within seconds [27–29].

The function of the dark eye markings in females is unknown, but its complete absence in males makes the explanations of correlated gene expression, camouflage and generally improved vision in shallow waters unlikely. This leaves us with four non-exclusive potential explanations for the dark eyes. First, that dark eyes are a female declaration of intent to mate. Second, that displaying dark eyes is a means by which a female can establish dominance over other females. Third, that males have a prior preference for dark eyes, which if displayed should make the female more attractive. Fourth, that dark eyes affect female vision and help her to accurately assess her mate.

Here we investigate the first three potential explanations and return to the fourth in the discussion. We first (A) attempt to characterize occasions when females voluntarily display dark eyes by allowing two females to freely associate with a nestholding male. We then experimentally test (B) if dark eyes play a role in female aggression and (C) if males have a preference for dark eyes. We formulated the following hypotheses: 1) If dark eyes declare an intention to spawn, we predict that the ornament will be more frequently displayed by rounder females, which are more gravid and closer to spawning, and primarily in conjunction with male courtship. 2) If dark eyes are used in intra-sexual competition, we predict that round females interacting aggressively, will display them. 3) If dark eyes correspond to a pre-existing male preference, we predict that males will prefer to associate with dark-eyed females.

## Materials and method

### Study species

The sand goby *Pomatoschistus minutus* is a small fish common in nearshore habitats along the coasts of northern Europe. During the breeding season, sand gobies have a reduced swim bladder leaving them less buoyant, and tend to hop along the substrate or rest on their fins, rather than swim higher up in the water column [36]. In early spring, adults migrate to sandy shallow bays to reproduce [37]. Parental care is a central aspect of sand goby reproduction and males construct nests by excavating a burrow beneath a mussel shell or a rock and covering it with sand swirled up with the tail [38]. Females place their eggs in a single layer in the ceiling of the nest and the male alone cares for the eggs until they hatch. Both sexes are polygamous. The male typically cares for eggs from several females in the nest and both sexes spawn repeatedly throughout the spring and summer [39–41]. Both males and females perform courtship routines before mating (described in more detail in [27] and [41]). The male displays darkened fins that also emphasize an iridescent blue spot on the dorsal fin and an iridescent blue band on the anal fin. Nest-holding males often venture approximately 10–50 cm from the nest to court females. With erect fins, the male performs a “lead swim” towards the nest and fans the nest in an exaggerated manner. If interested, females may follow the male and hover close to the nest [29,41]. At some point during the courtship, females may also develop conspicuously dark eyes, with the dark markings extending to the skin around the eyes and also down towards the mouth [27–29].

### Experimental design

The study was conducted at Kristineberg research station (Sven Lovén Centre for Marine Infrastructure, University of Gothenburg) on the Swedish west coast in May and June 2014 (A and C) and in May 2016 (B). Sand gobies were collected using a hand trawl in Bökevik bay near the station, separated by sex and placed in 130 L holding aquaria, furnished with a 3 cm layer of sand for fish to burrow in and supplied with running seawater. Fish in captivity were housed in a greenhouse (experiment A) or in a room with large windows (experiments B and C), allowing them to experience natural daylight hours. The fish in the holding aquaria were fed every third day with finely chopped frozen brown shrimp *Crangon crangon*, cod *Gadus morhua* and Alaska pollock *G*. *chalcogrammus*. All data can be found in the Supporting information.

#### (A) Free interaction study

Males were placed individually in experimental aquaria (39x22x25 cm). Each aquarium was furnished with a layer of sand and a halved PCP tube to serve as standardized nest site. To encourage nest building, a ripe female in a transparent plastic vial was put in front of the nest in each aquarium, and left for 12 hours. The vial was supplied with holes and a mesh net over the top to allow for water exchange. Before recording, an additional female of slightly different size was placed in the vial to acclimatize for 1 hour before they both were released and allowed to move freely. We judged that this acclimatization period would have mitigated any priority effect between the females. Male and female behaviors were recorded for one hour using a digital video camera. We obtained a total of 21 replicates.

From the video recording, the occurrence of females displaying dark eyes was observed (yes/no), along with the relative size (larger or smaller in terms of length) of the female displaying them, the events immediately preceding and following the instance of the darkened eyes, in particular whether there was any interaction with any of the other fish in the aquarium. Female roundness, as a measure of proximity to spawning, was scored in quarter-steps from 1–3 with 1 being very slim and 3 very round, and the eye color of any female that spawned was noted. The quality of the nest was scored on the scale of 1–3 with 1 being an unbuilt nest and 3 a fully built and covered nest. The intensity of male courtship was scored on a scale from 0–3 where 0 indicates no courtship and 3 very intense. It was not always possible to determine towards which female the male directed the courtship, and male courtship was therefore only scored for the replicate as a whole.

#### (B) Female aggression experiment

Mirrors have often been used to elicit aggressive intra-sexual responses in males (e.g. [42]). We used the same approach to test whether females display dark eyes in an intra-sexual aggressive context. Prior to the experiment, females from the storage aquaria were photographed beside a ruler and placed in an experimental aquarium (36x21x25 cm), alone (N = 9), in pairs (N = 10) or in groups of three (N = 6) in the evening, giving them the night to acclimatize. When more than one female were placed in an aquarium, it was always possible to visually distinguish them on the basis of size and roundness. The next day, a mirror (15x15 cm) was placed inside the aquarium along the side wall and adjacent to the front pane, after which activities were recorded for 20 minutes using a video camera. The females were then returned to a storage tank. From the recordings, we noted whether females engaged in aggressive behavior with the mirror and if they displayed dark eyes. Only interactions between a female and the mirror were counted and used in the analyses, as interactions between the females were either rare or not noticeable to the observer. We obtained 25 replicates.

#### (C) Male mate choice experiment

To test whether males prefer females with dark eyes, we performed a male mate choice experiment. Prior to the experiment, each experimental aquarium (75x21x24 cm) was divided into three approximately 25 cm long sections, demarcated by lines painted on the outside of the aquarium using a permanent marker. Each aquarium was furnished with a layer of sand and a halved clay flower pot, serving as a standardized nest site, was placed in the central section. A male and two stimuli females were introduced into each tank. The females were present to encourage nest building, but kept in a transparent plastic vial (same model as in A) to avoid further interactions. The stimuli females were removed once the males had built nests. For each replicate, two females of similar body size and roundness were anesthetized with MS-222 (70 mg MS-222/L seawater). Each female was dabbed dry around the snout and eyes and painted with either a black or a transparent non-toxic marker pen (100 “Black” and 0 ‘‘Colorless Blender”, Copic). The same kind of dye has previously been used to manipulate ventral ornaments of female two-spotted gobies *Gobiusculus flavescens* [43]. The painted females, each placed inside a transparent plastic vial in which they were fully visible throughout the experiment, were randomly assigned to either the left or the right hand side of the tank. A video camera was used to record activities in each replicate for 1200 seconds after which the females were euthanized using an overdose of MS-222 while the males were returned to the sea. From the video recordings, the total number of seconds the male spent beyond either demarcation line was measured, whereas time in the central zone was not. Because the time a male associates with a black-painted female is not independent from the time associated with the transparent-painted female, preference was calculated as the difference in time the male spent on either side, i.e. choice score = time_black_−time_transparent_. Thus, a strong preference for females manipulated to have black eyes would generate a score of 1200, a strong preference for females treated with the transparent marker would result in a score of -1200, whereas a score close to zero would either mean that the male had spent most time in the neutral zone, or that he had spent the same amount of time inspecting both females. A total of 20 replicates were obtained.

#### Statistics and data

Categorical results (dark eyes or not) were analyzed using logistic regressions with non-significant (p>0.05) interaction terms removed. The effect of nest quality and courtship intensity on displays of dark eyes were analyzed using Wilcoxon rank sum test. One-tailed t-test against an expected mean of 0 was used to analyze male mate preference. Data heteroscedasticity was assessed using Fligner-Killeen tests. R (v. 3.2.1) was used to perform the statistical analyses and to create the figures. All data are tabulated in Supplementary material.

#### Ethical approval

This study was carried out in accordance with national and international guidelines for the care and use of laboratory animals. Only fish that appeared healthy were used in the experiments and no fish died or were killed during the study. Ethical permission for the experimental procedures were obtained from the Swedish Animal Welfare Agency (interaction and female aggression studies: dnr 86–2013, male preference study: dnr 205–2013).

## Results

### (A) Free interaction study

**Fig 1:**
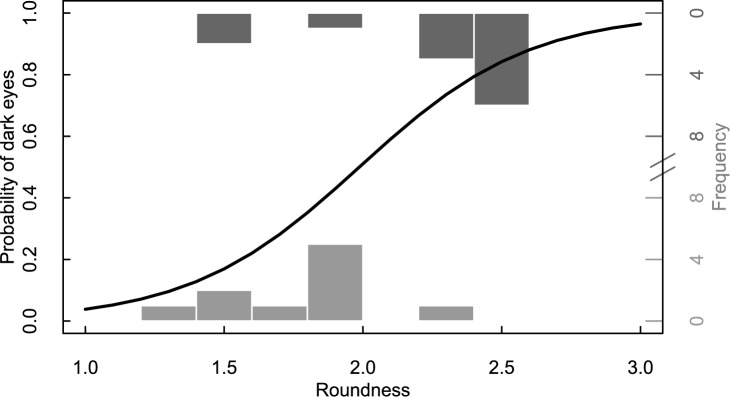
Bar chart illustrating the relation between female roundness (black curve, logistic regression of female roundness and dark eyes) and frequency of dark eyes in replicates where dark eyes were observed (dark grey bars: Females showing dark eyes N = 12, light grey bars: Females not showing dark eyes N = 10).

In 11 of the 21 replicates, at least one female displayed dark eyes at some point during the recording and in one of these both females displayed dark eyes, totaling 12 females displaying dark eyes. Some females that displayed dark eyes temporarily lost the coloration but later regained it, and 15 instances of dark eyes were observed in all. Furthermore, three matings were observed, all of which involved females with dark eyes. In the eleven replicates in which dark eyes were observed, females that displayed dark eyes had a higher average roundness score (2.23, range: 1.5–2.5) compared to females who did not (1.83, range: 1.25–2.0), whereas relative size did not matter (logistic regression: roundness z = 2.14, p = 0.032; relative size z = -0.083, p = 0.93; Fig 1). This result also holds true when the full data set is used (logistic regression: roundness z = 2.15, p = 0.032, relative size z = -0.23, p = 0.81). The larger female showed dark eyes in five replicates, the smaller female in a further five replicates, and in one replicate the females were of similar size.

In terms of events preceding a female displaying dark eyes, four instances included male-female interaction, typically courtship, and three instances female-female interaction, while the other instances did not appear to be part of any interaction. The instances of dark eyes were not followed by any apparent reaction from the male or the other female in the aquarium. Neither the mean courtship intensity by the male nor the mean nest quality score differed significantly between replicates that involved dark eyes and replicates that did not (Wilcoxon rank sum test: courtship: W = 77.5, p = 0.10; nest: W = 61.5, p = 0.64), though both scores were higher in replicates that involved dark eyes (courtship mean: 2.0, range: 0–3; nest mean: 2.5, range: 1–3) than in replicates that did not (courtship mean: 1.4; range: 0–3; nest mean: 2.3, range: 1–3). In three replicates, the male was observed to block repeated attempts by a female to enter the nests (in all cases the females showed dark eyes, in one replicate the female eventually mated).

### (B) Female aggression experiment

In most replicates, at least one female interacted aggressively with the mirror. In only 6 of the 25 replicates (five in one-female treatment and one in the two-female treatment), no interaction was seen between the female(s) and the mirror, while in the remainder interaction was recorded. Dark eyes were not observed in any replicate.

### (C) Male mate choice experiment

**Fig 2:**
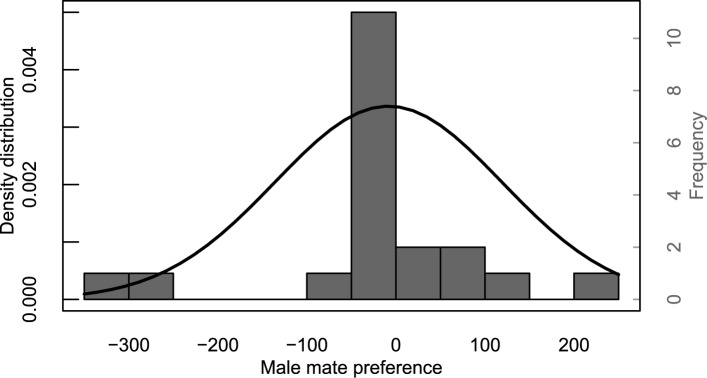
Frequency distribution for male mate preference (difference between time spent with female painted with black and female painted with transparent ink, N = 20). The associated normal density distribution (mean μ = -8.35, not significantly different from 0) shown as a black curve.

In eight out of the 20 replicates, the male remained inside the nest and thus received a choice score of zero. There was no significant effect of female eye color on the amount of time the male spent with either female (choice score), either when including all males (one-sample t-test against expected μ = 0: t_19_ = -0.315, p = 0.76; Fig 2) or just the active males (one-sample t-test against expected μ = 0: t_11_ = -0.310, p = 0.76).

## Discussion

In species where males compete over access to females, female ornamentation is often either absent or a drab version of the male ornamentation. This makes the habit of female sand gobies to display a conspicuous dark coloration around the eyes, which is completely absent in males, particularly intriguing. Female coloration is usually hypothesized to correspond either to a male preference, to female-female competition and dominance, or to represent a declaration of intent, showing the male that the female is ready to spawn. Here, we find that round females, carrying mature eggs, are more likely to show dark eyes than are slimmer females, but we find no evidence that males respond behaviorally to dark eyes or prefer dark-eyed females, or that dark eyes are displayed in aggressive intra-sexual interactions. Since dark eyes are associated with impending spawning, it may be an aspect of female mate choice, possibly a display intended to attract the attention of the male (without necessarily being successful). Alternatively, its function may be unrelated to communication, for example, it may be related to female vision.

### Dark eyes as a declaration of intent?

We found that round females were more likely to show dark eyes than slimmer females. Generally, both mate search and courtship carry risks of exposure and predation [44], and minimizing the effort spent on potential but unreceptive mates would be in the interest of both the male and the female. In species where females may inspect several males before deciding on a mate (as is the case in sand gobies; [28]), cues that indicate intent can act as an attention-seeking display to attract male attention and invite male courtship. It has been suggested that male sand gobies use courtship to attract female attention, whereas female mate choice is influenced by an array of cues other than courtship intensity [45]. It is thus possible that by signaling mating intent, females attract male attention and are offered better access to the male and the nest, which might speed up mate assessment. Furthermore, since male sand gobies are sometimes seen to block females trying to enter the nests, it is also possible that a nest-guarding male may assess female intent before allowing her into the nest, as this could pose a risk to any eggs already present in the nest. Notwithstanding, we found no evidence for males to respond behaviorally to dark eyes or prefer dark-eyed females. However, if dark eyes indeed declare spawning intent, possibly attracting the initial attention of a male, it would agree with our finding that rounder females are more likely to display dark eyes. Furthermore, the three females we observed spawning during the video recordings all had dark eyes, suggesting a link between the coloration and final mate choice. In contrast to traits indicating aspects of mate quality [46], attention seeking display does not necessarily carry costs or correlate with the quality of the signaler [7]. Hence, the dark eyed females may have attracted the male’s attention, but for some reason the males did not respond behaviorally, at least not in a way that we could quantify. Whether dark eyes do attract male attention needs further investigation.

### Dark eyes as an aspect of female aggression?

Body coloration has frequently been demonstrated to reflect dominance, mating status or competitive ability, in many species. The black badge in male house sparrows *Passer domesticus* is related to dominance [47] while the plumage of female red-winged blackbirds *Agelaius phoeniceus* is more conspicuous in populations where females help defend the territory compared to when only males are territorial [48]. In fish, color-associated aggression and dominance can be genetic or status-dependent. Color morph determines dominance among female *Neochromis omnicaeruleus* [49], while female convict cichlids *Amatitlania* spp. that develop a bright orange abdomen attract aggression from other females [50,51]. The purpose of our mirror experiment was to determine whether the display of dark eyes can be separated from the courtship context, i.e. if it may be displayed during female-female aggression without a male present. It should be noted that there were no resources for the females to compete for. However, we found no evidence that dark eyes play any part in female aggression even though we observed highly aggressive females. This is noteworthy given that a darkening of fins and body is a conspicuous aspect of aggression in male sand gobies [41].

### Dark eyes in response to male preference?

Bright coloration in females may correspond to male mate preference, although female ornaments are not always clearly distinguishable from phenotypic benefits [15]. For instance, in bluethroats *Luscinia s*. *svecica*, males prefer colorful females [52], even though female coloration appears to be unrelated to parental qualities [53,54]. On the other hand, in the two-spotted goby *Gobiusculus flavescens*, males prefer colorful females [43], which also lay larger clutches [55]. Here, however, we found no indication that male sand gobies preferred dark-eyed females. The vials used in our experimental set-up did not allow females much freedom of movement and although the activity level of the females was not particularly variable, we cannot exclude the possibility that female behavior may have influenced the results. Another explanation may be that males assess potential partners based on behavior as well as other preferred attributes such as roundness and larger size. Since behavior is more malleable than physique, a small or relatively slim female willing to mate might be sufficiently attractive to the male if she declares intent by showing dark eyes. Since the females in experiment (C) were size-matched, this aspect could not be tested here, however, in experiment (A) rounder females were more likely to display dark eyes than slimmer ones, while there was no difference between larger and smaller females. It is also possible that males did not respond to the artificially darkened eyes as they would have done to naturally displayed dark eyes. Even so, the evidence from experiment (A) did not indicate that naturally displayed dark eyes elicit any obvious response in males.

### Dark eyes as a means of improving visual accuracy?

Eye color has been suggested to act as an anti-glare and could therefore hypothetically improve the accuracy of visual assessment. For instance, many American football and baseball players paint a black stripe underneath their eyes (so called ‘eye black’), to reduce glare and improve vision [56]. Similarly, in Arctic reindeer *Rangifer tarandus*, the color of the *tapetum lucidum* (a thin tissue layer behind the retina) changes over the seasons, which may adaptively adjust the retinal sensitivity [57]. This phenomenon appears to have received most attention in mammals, and it is open to speculation whether it also applies to fish. If it does, the timing of the dark eyes display that we found here, just prior to spawning or by very round females presumably close to spawning, suggests that such improved vision is likely to be associated with mate evaluation. Given that sand gobies normally adjust their body color, including eye color, to the background for camouflage [58], the dark eyes in females appear conspicuous and the temporary nature of the coloration may be an adaptation to heightened predation risk. Given that most breeding takes place in shallow and sandy bays, glare is likely to be pronounced. It is therefore possible that the dark eyes aid ready-to-spawn females in their assessment of potential mates, and although it is obviously premature to conclude whether dark eyes serve this function, it also should not be ruled out.

In conclusion, dark eyes in sand goby females are associated with readiness to spawn, but the exact function of this temporary shown color change is not fully understood. It is not shown during female-female aggression and it also appears to be unrelated to male mate choice. It is, however, possible that it is an aspect of female mate choice, displayed as part of female courtship behavior towards the male. Alternatively, it may have a completely different function, possibly related to vision. Future research is needed to tell which, if any, of these suggested functions play a role.

## Supporting information

S1 Appendix Raw data collected on male and female behaviour. (DOCX)
