# Supplementary material for: Dark eyes in female sand gobies indicate readiness to spawn
Source: PLoS One. 2017 Jun 7;12(6):e0177714. doi: 10.1371/journal.pone.0177714 (PMC5462351; doi:10.1371/journal.pone.0177714)
Supplement: S1 Appendix — (DOCX) [file pone.0177714.s001.docx]

**Supplementary Information**

S1 Table. **Voluntary female display of dark eyes.** Male courtship and nest quality, females displaying dark eyes and preceding interaction, female relative size and roundness and mating.

| Replicate | Male courtship (0-3) | Nest quality (1-3) | Replicate dark eyes  (y/n) | Female | Female dark eyes (y/n) | Preceding interaction | Largest female (y/n) | Female roundness | Mated  (y/n) |
| --- | --- | --- | --- | --- | --- | --- | --- | --- | --- |
| 1 | 3 | 3 | y | F1 | y | F1-F2 | y | 2.5 | y |
|  |  |  |  | F2 | y | F1-F2 | n | 2.5 |  |
| 2 | 3 | 2 | y | F1 | n |  | n | 2 |  |
|  |  |  |  | F2 | y | M-F1F2 | y | 2 |  |
| 3 | 1 | 3 | n | F1 | n |  | y | 2.25 |  |
|  |  |  |  | F2 | n |  | n | 2.25 |  |
| 4 | 2 | 3 | y | F1 | y | M-F1 | y | 2.5 | y |
|  |  |  |  | F2 | n |  | n | 1.75 |  |
| 5 | 0 | 1 | n | F1 | n |  | n | 2 |  |
|  |  |  |  | F2 | n |  | y | 2 |  |
| 6 | 2 | 1 | n | F1 | n |  | y | 2.5 |  |
|  |  |  |  | F2 | n |  | n | 2.25 |  |
| 7 | 0 | 1 | y | F1 | n |  | y | 2.25 |  |
|  |  |  |  | F2 | y |  | n | 1.5 |  |
| 8 | 3 | 3 | n | F1 | n |  | y | 2 |  |
|  |  |  |  | F2 | n |  | n | 2 |  |
| 9 | 2 | 3 | y | F1 | n |  | same | 1.5 |  |
|  |  |  |  | F2 | y | none | same | 2.25 |  |
| 10 | 3 | 3 | y | F1 | n |  | y | 2 |  |
|  |  |  |  | F2 | y; y | M-F1F2; none | n | 2.25 | y |
| 11 | 2 | 1 | y | F1 | y; y | none; none | y | 1.5 |  |
|  |  |  |  | F2 | n |  | n | 1.25 |  |
| 12 | 2 | 2 | n | F1 | n |  | y | 2 |  |
|  |  |  |  | F2 | n |  | n | 2.25 |  |
| 13 | 1 | 2 | n | F1 | n |  | y | 2.5 |  |
|  |  |  |  | F2 | n |  | n | 1.5 |  |
| 14 | 2 | 3 | y | F1 | n |  | y | 2 |  |
|  |  |  |  | F2 | y | none | n | 2.5 |  |
| 15 | 2 | 2 | y | F1 | y | none | y | 2.25 |  |
|  |  |  |  | F2 | n |  | n | 1.5 |  |
| 16 | 1 | 3 | y | F1 | y | none | y | 2.5 |  |
|  |  |  |  | F2 | n |  | n | 2 |  |
| 17 | 2 | 3 | n | F1 | n |  | n | 1.75 |  |
|  |  |  |  | F2 | n |  | y | 2 |  |
| 18 | 1 | 3 | n | F1 | n |  | n | 1.5 |  |
|  |  |  |  | F2 | n |  | y | 2 |  |
| 19 | 1 | 2 | n | F1 | n |  | y | 2.25 |  |
|  |  |  |  | F2 | n |  | n | 2 |  |
| 20 | 2 | 3 | y | F1 | n |  | y | 2 |  |
|  |  |  |  | F2 | y; y | F1-F2; M-F1F2 | n | 2.5 |  |
| 21 | 1 | 3 | n | F1 | n |  | y | 2.25 |  |
|  |  |  |  | F2 | n |  | n | 2 |  |

S2 Table. **Female aggression towards mirror image.** Number of aggressive interactions with mirror image for each female, number of females and female length for each replicate.

| Replicate | No. of females | Length (mm) | Interactions with mirror |
| --- | --- | --- | --- |
| 1 | 2 | 72 | 39 |
|  |  | 57 | 0 |
| 2 | 1 | 67 | 0 |
| 3 | 1 | 68 | 4 |
| 4 | 2 | 55 | 1 |
|  |  | 72 | 43 |
| 5 | 1 | 50 | 29 |
| 6 | 2 | 55 | 0 |
|  |  | 70 | 8 |
| 7 | 2 | 61 | 0 |
|  |  | 52 | 0 |
| 8 | 2 | 60 | 8 |
|  |  | 60 | 59 |
| 9 | 1 | 58 | 1 |
| 10 | 2 | 53 | 7 |
|  |  | 57 | 3 |
| 11 | 1 | 57 | 0 |
| 12 | 1 | 62 | 0 |
| 13 | 1 | 56 | 0 |
| 14 | 2 | 72 | 3 |
|  |  | 60 | 1 |
| 15 | 1 | 57 | 0 |
| 16 | 1 | 57 | 36 |
| 17 | 2 | 60 | 0 |
|  |  | 56 | 2 |
| 18 | 2 | 72 | 97 |
|  |  | 61 | 8 |
| 19 | 2 | 56 | 16 |
|  |  | 60 | 1 |
| 20 | 3 | 62 | 34 |
|  |  | 60 | 103 |
|  |  | 56 | 4 |
| 21 | 3 | 56 | 21 |
|  |  | 57 | 13 |
|  |  | 60 | 10 |
| 22 | 3 | 62 | 40 |
|  |  | 59 | 12 |
|  |  | 57 | 0 |
| 23 | 3 | 62 | 1 |
|  |  | 50 | 49 |
|  |  | 64 | 1 |
| 24 | 3 | 63 | 15 |
|  |  | 71 | 26 |
|  |  | 50 | 1 |
| 25 | 3 | 66 | 15 |
|  |  | 55 | 0 |
|  |  | 73 | 17 |

S3 Table. **Male mate choice data.** Seconds the focal male spent with female painted black and female painted transparent.

|  | Seconds spent with female | | |
| --- | --- | --- | --- |
| Male | Black | Transparent | Mate choice |
| 1 | 0 | 0 | 0 |
| 2 | 0 | 0 | 0 |
| 3 | 413 | 283 | 1300 |
| 4 | 0 | 0 | 0 |
| 5 | 0 | 0 | 0 |
| 6 | 30 | 0 | 30 |
| 7 | 0 | 24 | -24 |
| 8 | 98 | 119 | -21 |
| 9 | 0 | 32 | -32 |
| 10 | 241 | 9 | 232 |
| 11 | 0 | 0 | 0 |
| 12 | 0 | 0 | 0 |
| 13 | 79 | 147 | -68 |
| 14 | 61 | 8 | 53 |
| 15 | 71 | 24 | 47 |
| 16 | 81 | 0 | 81 |
| 17 | 14 | 340 | -326 |
| 18 | 0 | 0 | 0 |
| 19 | 0 | 269 | -269 |
| 20 | 0 | 0 | 0 |
